# Meningitis patients with *Angiostrongylus cantonensis* may present without eosinophilia in the cerebrospinal fluid in northern Vietnam

**DOI:** 10.1371/journal.pntd.0008937

**Published:** 2020-12-22

**Authors:** Tomoko Hiraoka, Ngo Chi Cuong, Sugihiro Hamaguchi, Mihoko Kikuchi, Shungo Katoh, Le Kim Anh, Nguyen Thi Hien Anh, Dang Duc Anh, Chris Smith, Haruhiko Maruyama, Lay-Myint Yoshida, Do Duy Cuong, Pham Thanh Thuy, Koya Ariyoshi

**Affiliations:** 1 Department of Clinical Medicine, Institute of Tropical Medicine (NEKKEN), Nagasaki University, Nagasaki, Japan; 2 Department of Clinical Tropical Medicine, Nagasaki University Graduate School of Biomedical Sciences, Nagasaki, Japan; 3 Department of Infectious Diseases, Bach Mai Hospital, Hanoi, Vietnam; 4 Department of General Internal Medicine, Fukushima Medical University, Fukushima, Japan; 5 Department of Immunogenetics, Institute of Tropical Medicine (NEKKEN), Nagasaki University, Nagasaki, Japan; 6 Department of General Internal Medicine, Nagasaki Rosai Hospital, Nagasaki, Japan; 7 Vietnam Research Station, Institute of Tropical Medicine (NEKKEN), Nagasaki University, Hanoi, Vietnam; 8 National Institute of Hygiene and Epidemiology, Hanoi, Vietnam; 9 Department of Global Health, School of Tropical Medicine and Global Health, Nagasaki University, Nagasaki, Japan; 10 Department of Clinical Research, London School of Hygiene and Tropical Medicine (LSHTM), London, United Kingdom; 11 Department of Infectious Diseases, Division of Parasitology, Faculty of Medicine, University of Miyazaki, Miyazaki, Japan; 12 Department of Pediatric Infectious Diseases, Institute of Tropical Medicine (NEKKEN), Nagasaki University, Nagasaki, Japan; 13 Infection Prevention and Control, The Partnership for Health Advancement in Vietnam (HAIVN), Hanoi, Vietnam; PUCRS, BRAZIL

## Abstract

**Background:**

Eosinophilic meningitis (EM) is a rare clinical syndrome caused by both infectious and noninfectious diseases. In tropical pacific countries, *Angiostrongylus cantonensis* is the most common cause. However, the EM definition varies in the literature, and its relation to parasitic meningitis (PM) remains unclear.

**Methodology/Principal findings:**

Adult and adolescent patients of 13 years old or above with suspected central nervous system (CNS) infections with abnormal CSF findings were prospectively enrolled at a tertiary referral hospital in Hanoi, Vietnam from June 2012 to May 2014. Patients with EM or suspected PM (EM/PM) were defined by the presence of either ≥10% eosinophils or an absolute eosinophil cell counts of ≥10/mm^3^ in the CSF or blood eosinophilia (>16% of WBCs) without CSF eosinophils. In total 679 patients were enrolled: 7 (1.03%) had ≥10% CSF eosinophilia, 20 (2.95%) had ≥10/mm^3^ CSF eosinophilia, and 7 (1.03%) had >16% blood eosinophilia. The patients with ≥10% CSF eosinophilia were significantly younger (p = 0.017), had a lower body temperature (p = 0.036) than patients with ≥10/mm^3^ CSF eosinophilia among whom bacterial pathogens were detected in 72.2% (13/18) of those who were tested by culture and/or PCR. In contrast, the characteristics of the patients with >16% blood eosinophilia resembled those of patients with ≥10% CSF eosinophilia. We further conducted serological tests and real-time PCR to identify *A*. *cantonensis*. Serology or real-time PCR was positive in 3 (42.8%) patients with ≥10% CSF eosinophilia and 6 (85.7%) patients with >16% blood eosinophilia without CSF eosinophils but none of patients with ≥10/mm^3^ CSF eosinophilia.

**Conclusions:**

The etiology of PM in northern Vietnam is *A*. *cantonensis*. The eosinophil percentage is a more reliable predictor of parasitic EM than absolute eosinophil count in the CSF. Patients with PM may present with a high percentage of eosinophils in the peripheral blood but not in the CSF.

## Author summary

Eosinophilic meningitis (EM) is a rare meningitis accompanied by eosinophils in the CSF and caused by multiple etiologies. *Angiostrongylus cantonensis*, which is a rat lungworm parasite, is the most common cause in tropical Asia. Previous papers have defined EM as CSF eosinophils ≥10% or CSF eosinophils ≥10/mm^3^. However, the relationship of EM to parasitic meningitis (PM) remains unclear. This prospective study enrolled 679 patients with suspected CNS infection who were admitted to a tertiary referral hospital in Hanoi, Vietnam from June 2012 to May 2014. The characteristics of patients with ≥10% CSF eosinophilia resembled those of patients with >16% blood eosinophilia without CSF eosinophils, whereas those of patients with ≥10/mm^3^ CSF eosinophilia were comparable with those of patients with typical bacterial meningitis. Serology or real-time PCR for *A*. *cantonensis* was positive in 3 out of 7 patients with ≥10% CSF eosinophilia and 6 out of 7 patients with > 16% blood eosinophilia without CSF eosinophils but none of patients with ≥10/mm^3^ CSF eosinophilia. The percentage, in contrast to the absolute eosinophil count in CSF, is reliable for predicting parasitic EM. Patients with PM may present with eosinophilia in the peripheral blood but not in the CSF.

## Introduction

Eosinophilic meningitis (EM) is a rare clinical syndrome characterized by meningeal inflammation and eosinophilic pleocytosis in the cerebrospinal fluid (CSF) [1,2]. The first case of EM was reported in Taiwan in 1945. In this case, eosinophilia of the CSF and peripheral blood was observed, and then *Angiostrongylus cantonensis* larvae were identified in the CSF [3]. Since this report, EM cases have been recognized and reported in the Pacific Ocean islands, East Asia, and North America [4–6]. There are various etiologies of CSF eosinophilia, including parasitic infections of the central nervous system (CNS) and other infectious diseases, such as tuberculous meningitis, cerebrospinal syphilis, viral and fungal meningitis, as well as noninfectious causes, such as drug allergies, multiple sclerosis and neoplasms, for example, Hodgkin’s disease or leukemia [1,2,5]. However, the most common etiologies in Southeast Asia and other tropical countries are parasitic infectious diseases, especially *A*. *cantonensis*, *Gnathostoma spinigerum*, cysticercosis (*Taenia solium*) and *Toxocara canis* [7]. Therefore, in countries with tropical climates, it is important to determine whether meningitis is parasitic meningitis (PM) because specific treatment is required [7].

The definition of EM varies. Many EM publications have followed the definition originally suggested by Kuberski [8]; the presence of at least 10% eosinophils in the total CSF white blood cell (WBC) count or the presence of at least 10 eosinophils/mm^3^ in the CSF [2]. However, this criterion was based on a limited observation of 123 CSF samples derived from 110 pediatric patients with a variety of clinical diagnoses in Hawaii [8]. Furthermore, another definition of EM was also suggested by Punyagupta et al. [9]; patients with an acute headache of fewer than 2 months with a CSF WBC count/mm^3^ of 20 or more, of which 10% or more are eosinophils [10]. The author used this criterion to select 484 patients with probable angiostrongyliasis in Thailand. Many papers from Asia, especially Thailand, have followed this criterion [11,12].

In addition, some EM papers have reported that 30–80% of patients with meningitic angiostrongyliasis have accompanying blood eosinophilia [4,12,13]. Swanyawisuth et al. [14] discussed the significance of peripheral eosinophilia as an indicator of meningitic angiostrongyliasis. They found that if patients with suspected PM had an eosinophil count of more than 798 cells in their peripheral blood, the sensitivity and specificity of meningitis due to *A*. *cantonensis* reached 76.6% and 80.2%, respectively. This group investigated the presence of PM with a blood serological test without lumbar puncture. Schulte et al. [15] also reported a positive predictive value for helminth infections of 46.6% among travelers returning from tropical countries with blood eosinophilia > 16% of the WBC count.

To our knowledge, however, few studies have systematically attempted to delineate the clinical implication of various definitions of EM to date with the objective of identifying PM [5,16]. We believe that describing the clinical characteristics of patients with EM or suspected PM (EM/PM) classified by each definition and confirming the causative parasites will provide useful information to clinicians to improve clinical judgment and management. To improve the clinical management of PM in northern Vietnam, we conducted a prospective study of CNS infection in this area. The primary objectives of this study were to investigate the epidemiological and clinical characteristics of various definitions of EM/PM in relation to the pathogenic parasite. The secondary objective was to further understand the value of current definitions of EM for predicting PM.

## Methods

### Ethics statement

This study was approved by the independent ethics committees of the Institute of Tropical Medicine, Nagasaki University (approval number: 12021085–4), Nagasaki, Japan, Bach Mai Hospital and the National Institute of Hygiene and Epidemiology as part of a “Collaborative Study on Emerging and Re-emerging Infectious Diseases in Vietnam” (approval number: 15-IRB, 2011), Hanoi, Vietnam. Written informed consent was obtained from all patients prior to enrollment. For those who were unconscious, a parent or guardian was asked to provide informed consent, and the data were analyzed anonymously.

### Study design and setting

Between June 2012 and May 2014, we conducted a prospective observational study of undiagnosed febrile illness in the Infectious Disease Department of Bach Mai Hospital, which is the largest government referral medical center in Hanoi covering patients in northern Vietnam, as published previously [17].

### Inclusion and exclusion criteria and case definition

Patients were enrolled according to the following criteria: 1) age ≥ 13 years, 2) axillary temperature > 37.5°C (any time from onset to admission), and 3) lumbar puncture due to suspected CNS infection by the admitting physician. The exclusion criteria were patients with a clinically definitive diagnosis (e.g., malaria, dengue fever, mumps, food-related diarrhea, cellulitis, animal bite), patients with hepatitis-related disease (e.g., viral hepatitis, alcoholic liver disease, autoimmune hepatitis, cirrhosis, liver cancer), and patients with microbiologically identified infectious diseases (e.g., already diagnosed at referral hospitals). Regarding fever criteria, even if patients did not have fever at admission, they were enrolled as long as they had had fever at any time point from onset to admission. Patients and samples to be enrolled were determined on the following morning of each admission day.

We defined abnormal CSF as CSF protein > 0.4 g/l and CSF absolute WBC count > 5/mm^3^. At Bach Mai Hospital, we defined EM/PM cases in three ways: 1) the eosinophils accounted for ≥ 10% of the total WBCs in the CSF and the absolute number of eosinophils was ≥ 10/mm^3^ in the CSF, 2) the percentage of eosinophils was < 10% of the total WBC count in the CSF and the absolute number of eosinophils was ≥ 10/mm^3^ in the CSF, or 3) the absolute number of eosinophils was < 10/mm^3^ in the CSF and the percentage of eosinophils was > 16% of the peripheral blood WBC count.

### Data and sample collection

We prospectively collected epidemiological data (age, gender, place of occurrence, occupation, medical history, duration of fever, clinical presentation), and biological blood and CSF data. We also collected initial blood samples (plasma and buffy coat) and initial CSF samples after admission. At the hospital, the WBC type and number of cells in the CSF sample were confirmed by Giemsa staining when the total WBC count was greater than 10/mm^3^. After identifying patients with suspected EM or PM, we conducted a retrospective chart review to obtain further clinical information using hospital records.

### Biological analysis

#### Serological tests

We tested initial blood plasma samples of suspected EM/PM patients and 20 control patients for anti-parasite antibodies in enzyme-linked immunosorbent assay (ELISA). The antigen tested were those of *A*. *cantonensis*, *Toxocara canis*, *Paragonimus* spp., and *Strongyloides stercoralis*. According to internal data of Miyazaki University, the sensitivity / specificity of parasite ELISA was 90.0 / 99.1 for strongyloidiasis, 97.1 / 97.4 for paragonimiasis, 97.3 / 74.6 for larva migrant syndrome due to *Toxocara* or *Ascaris* infections. But the sensitivity / specificity of *A*. *cantonensis* has not yet been fully established due to lack of positive confirmed cases in Japan. The current ELISA for testing antibodies to *A*. *cantonensis* was based on a previously described method [18], in which *A*. *cantonensis* antigen was prepared from fourth-stage larvae recovered from the brains of experimentally infected rats. The sensitivity and specificity of this test were reported as 100% and 66.8%, respectively [19]. The 20 control patients were randomly selected from the list of non-EM patients in the same study, including patients with normal CSF (n = 8) and with abnormal CSF (bacterial meningitis: n = 5, tuberculosis (TB) meningitis: n = 1, and aseptic meningitis patients: n = 6).

#### Real-time polymerase chain reaction (PCR) for *A*. *cantonensis*

We conducted real-time PCR analyses for *A*. *cantonensis*, which is the most common parasite specie causing EM in Asia, using CSF samples from EM/PM patients fulfilling either of the three criteria. First, we prepared 200 mm^3^ of the CSF samples, as previously described [20], and then we extracted DNA from the samples using a QIAmp DNA Mini kit (QIAGEN, Hilden, Germany) with 100 mm^3^ of elution buffer. We performed TaqMan Real-time PCR for *A*. *cantonensis* with positive and negative controls for each assay.

For the real-time PCR for *A*. *cantonensis*, we followed the protocol of Qvarnstrom et al. [21], using TaqMan Universal Master Mix II (Thermo Fisher Scientific, Waltham, MA, USA) and an Applied Biosystems 7500 Real Time PCR system (Applied Biosystems, Foster City, CA, USA). A positive control was prepared from the whole worm body of *A*. *cantonensis*. We cut 3 worms into small pieces and extracted whole DNA from the worms using a QIAmp DNA Mini kit (QIAGEN, Hilden, Germany) with 100 mm^3^ of elution buffer. The upper limit of dilution of the positive control was 100,000 times for detection by TaqMan real-time PCR for *A*. *cantonensis*.

For the real-time PCR for *G*. *spinigerum*, we designed two sets of oligonucleotide primers to amplify a 144-bp fragment of the first internal transcribed spacer (ITS1) gene of *G*. *spinigerum* and a 115-bp fragment of the second internal transcribed spacer (ITS2) gene of *G*. *spinigerum*, based on Primer3 <https://primer3plus.com/cgi-bin/dev/primer3plus.cgi> with GenBank accession no. AB181155. The primers targeting ITS1 were Gspi-ITS1F (5’-CATCGGCTCTGATCTTCGCT-3’) and Gspi-ITS1R (5’-AGACACCAACGGATGCTGTT-3’); the primers targeting ITS2 were Gspi-ITS2F (5’-CATTCATCGAGCGGCAAGTG-3’) and Gspi-ITS2R (5’-GCGTACGCACCTCGATAAGA-3’). After confirming that the *G*. *spinigerum* positive control showed a single band by conventional PCR with each of the two sets of primers, using GoTaq Flexi DNA Polymerase (Promega Corporation, Madison, WI, USA), we performed SYBR Green Real-time PCR for *G*. *spinigerum* using each of the two sets of primers with Power SYBR Green PCR Master Mix (Thermo Fisher Scientific, Waltham, MA, USA) and a 7500 Real Time PCR System (Applied Biosystems, Foster City, CA, USA). The positive control for *G*. *spinigerum* was its whole genome, which was kindly provided by the Department of Helminthology, Faculty of Tropical Medicine, Mahidol University. The whole-genome DNA concentration was 3100 ng/mm^3^. The upper limit of the positive control dilution was 1,000,000 times for detection by SYBR Green Real-time PCR.

### Statistical analysis

We showed the demographic and clinical characteristics of each EM/PM group with those of the other patients using frequencies and percentages for categorical values and the median and interquartile range (IQR) for continuous variables. When we were comparing CSF eosinophils ≥ 10% group with other definition group individually or comparing the EM/PM criteria not fulfilled group with each definition group individually, categorical variables were compared by Fisher’s exact test, and continuous variables were compared by the Mann-Whitney nonparametric test. We calculated the odds ratio (OR) with 95% confidence intervals using logistic regression analysis. In addition, we calculated p-values among the 4 groups by Kruskal-Wallis test for continues variables and Chi-square test for categorical variables. Statistical analysis was conducted using STATA version 15 (StataCorp LLC, College Station, TX77845 USA). All tests were two-tailed, and p < 0.05 was considered statistically significant.

## Results

During the study period, from June 2012 to May 2014, 7,505 patients were admitted to the department, and 2,458 patients were hospitalized with undiagnosed febrile illness. Among them, 679 patients underwent lumbar puncture and were enrolled. Abnormal CSF was found in 431 (63.5%) patients. Blood samples of all patients (100%) were available, and CSF samples of 473 patients (69.7%) were available for this study.

**Fig 1:**
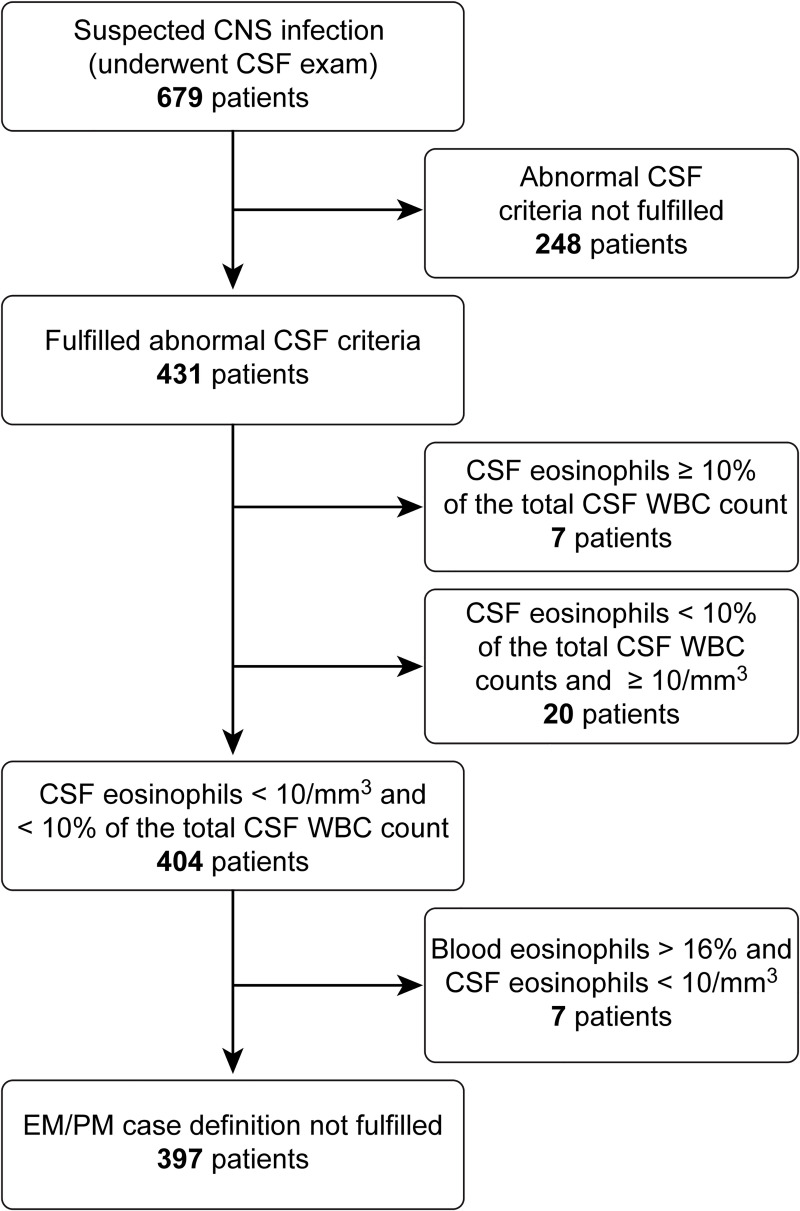
Flow chart of the patients enrolled in this study. CNS: central nervous system, CSF: cerebrospinal fluid, EM/PM: eosinophilic meningitis or suspected parasitic meningitis.

Among the 431 patients, 7 (1.03% of 679 patients) had eosinophils accounting for ≥ 10% of the total WBC count in the CSF, all of whose absolute eosinophil counts in the CSF were ≥ 10/mm^3^, and 20 (2.95% of 679 patients) had an absolute eosinophil count in the CSF ≥ 10/mm^3^ but eosinophils not accounting for ≥ 10%. Seven (1.03% of 679 patients) had eosinophils accounting for > 16% of the peripheral blood WBCs but no eosinophil in the CSF. The remaining 397 were non-EM/PM patients, who did not fulfill any EM/PM definition criteria. This included 22 patients who were diagnosed with TB meningitis as defined by positive result of TB-PCR test using CSF sample. There were no patients who had < 10/mm^3^ eosinophils in the CSF and eosinophils accounting for ≥ 10% of the total WBC count in the CSF. (Figs 1 and S1).

Among the 431 patients with abnormal CSF, the median (IQR) age was 44 (30–57) years old, male sex was predominant (n = 271, 62.9%), the median (IQR) duration of fever before admission was 6.5 (3–11) days, most patients had a headache (n = 385, 89.3%), and 33.4% (n = 143) had a Glasgow Coma Score (GCS) of ≤14. All patients had mild leukocytosis (median WBC 180/mm^3^, IQR 60–600, ranging from 6 to 31820) and elevated protein concentration (median 1.12 g/l, IQR 0.66–2.23, ranging from 0.41 to 16) in their CSF.

The characteristics and laboratory findings of patients are summarized according to each definition of EM/PM in Table 1 where p-values of four groups comparison are also shown. The median age of the CSF eosinophils ≥ 10% group was similar to that of the blood eosinophils > 16% group. Compared with the CSF eosinophils ≥ 10/mm^3^ group or the non-EM/PM group, the CSF eosinophil ≥ 10% group was significantly younger (p = 0.02, p = 0.0053, respectively) and tended to have a longer fever duration (p = 0.09, p = 0.3456, respectively). In addition, compared with the non-EM/PM group, the CSF eosinophil ≥ 10/mm^3^ group had a significantly shorter duration of fever prior to admission (p < 0.001). The median body temperature in the CSF eosinophils ≥ 10% and blood eosinophils > 16% groups was almost the same, whereas the body temperature of the CSF eosinophils ≥ 10/mm^3^ group was significantly higher than that of the CSF eosinophils ≥ 10% group (p = 0.036). Consciousness disorder (GCS < 15) was significantly higher in the CSF eosinophil ≥ 10/mm^3^ group (OR 3.8 [95% CI 1.5; 9.7], p = 0.006) compared with that in the non-EM/PM group.

**Table 1:** Characteristics and laboratory findings of patients in each EM/PM definition group.

Characteristics of patient0073	CSF eosinophils ≥10%, N = 7		CSF eosinophils ≥10/mm^3^, N = 20		Blood eosinophils >16%, N = 7		EM/PM definition not fulfilled, N = 397		P-value**
Age, years	29	[21–33]	44	[30–56]	32	[28–53]	45	[30–58]	0.0294
Male	5	(71.4)	15	(75.0)	6	(85.7)	246	(62.0)	0.364
**Symptoms and signs**									
Fever duration before admission, days (n = 430)	15	[2–20]	3	[2–3.5]	10	[5–25]	7	[3–11.5]	0.0006
BT,°C (n = 429)	37.3	[36.8–38]	38.3	[37.3–38.6]	37.5	[37–38]	38	[37.1–38.5]	0.1291
GCS < 15 (n = 428)	0	(0.0)	13	(65.0)	0	(0.0)	130	(33.0)	0.001
**CSF findings**									
WBC count, /mm3	1,380	[1,070–2,310]	5,585	[3,15–10,370]	420	[310–540]	142	[50–480]	0.0001
Neutrophils > 80%	0	(0.0)	19	(95.0)	0	(0.0)	69	(17.4)	< 0.0001
Eosinophils, % (n = 430)	45	[30–58.4]	0.95	[0.5–2.55]	0	[0–0]	0	[0–0]	0.0001
Eosinophil count, /mm3 (n = 430)	432	[284–1,349]	69.3	[36.2–121.5]	0	[0–0]	0	[0–0]	0.0001
Protein ≥1.00 g/l	3	(42.9)	19	(95.0)	5	(71.4)	207	(52.1)	0.002
Glucose ≤ 0.04 g/dl	0	(0.0)	6	(30.0)	0	(0.0)	26	(6.6)	0.001
Culture or bacterial PCR positive/performed (%)	1/7	(14.3)	13/18	(72.2)	0/5	(0.0)	67/376	(17.8)	< 0.0001
Real-time PCR for *A*. *cantonensis* positive / performed (%)	2/5	(40.0)	0/17	(0.0)	1/3	(33.3)	0/12#	(0.0)	0.008
**Peripheral blood findings**									
WBC count, /mm3	10,280	[8,820–10,890]	18,015	[10,075–23,700]	11,800	[9,120–16,500]	11,080	[7,960–15,30]	0.0134
Neutrophils, %	46.9	[39.2–58.1]	89.2	[81.7–92.5]	47.5	[41.6–56.1]	77.3	[67.6–85.4]	0.0001
Eosinophils, %	18.4	[10.3–27.1]	0	[0–0.1]	22.8	[18.3–30.1]	0.2	[0–1.1]	0.0001
Eosinophil count, /mm3	1,910	[900–2,670]	0	[0–10]	3,445	[1,540–4,760]	20	[0–100]	0.0001
Culture positive/performed (%)	0/1	(0.0)	5/17	(29.4)	0/1	(0.0)	38/258	(14.7)	0.369
ELISA for *A*. *cantonensis* positive / performed (%)	2/7	(28.6)	0/20	(0.0)	5/7	(71.4)	0/12#	(0.0)	< 0.0001
**Treatment**									
Antihelminthic medicine	7	(100.0)	0	(0.0)	3	(42.9)	4	(1.0)	< 0.0001
Antibiotics	4	(57.1)	20	(100.0)	6	(85.7)	388	(97.7)	< 0.0001
Other antimicrobial medicine*	0	(0.0)	4	(20.0)	1	(14.3)	112	(28.2)	0.268
Steroid	7	(100.0)	19	(95.0)	7	(100.0)	218	(54.9)	< 0.0001
**Outcome at discharge**									
Full recovery	2	(42.9)	5	(25.0)	1	(14.3)	77	(19.4)	0.836
No full recovery	5	(71.4)	15	(75.0)	6	(85.7)	319	(80.4)	0.836
Death	0	(0.0)	0	(0.0)	0	(0.0)	1	(0.2)	0.993

Values are given as actual counts (%) for categorical variables or medians [interquartile ranges] for continuous variables.

CSF: cerebrospinal fluid, EM/PM: eosinophilic meningitis or suspected parasitic meningitis, BT: body temperature, GCS: Glasgow coma scale, WBC: white blood cell, PCR: polymerase chain reaction.

*Antimicrobial medicine: Anti-TB medicine, Antiviral medicine and Antifungal medicine

**p-value are presented for comparison of parameters among this 4 groups: statistical tests include Kruskal-Wallis test and Chi-square test

#This does not include 8 control patients with normal CSF finding who were negative for real-time PCR and ELISA.

Comparisons of the four groups showed a significant difference in all laboratory findings except for blood culture result. This was because the characteristics of the CSF eosinophils ≥ 10/mm^3^ group was unique. The WBC counts, neutrophil percentages and CSF protein levels were higher, and the CSF glucose concentration was lower in the CSF eosinophil ≥ 10/mm^3^ group than the other groups. In this group, 19 (95%) patients had neutrophils accounting for more than 80% of the CSF WBCs, though in the CSF eosinophil ≥ 10% and blood eosinophil > 16% groups, no patient had neutrophils dominant in their CSF. Nineteen (95%) patients with CSF eosinophils ≥ 10/mm^3^ had more than 1.00 g/l CSF protein, and 6 (30%) patients had less than 0.04 g/dl CSF glucose, whereas none of patients with the CSF eosinophils ≥ 10% and blood eosinophils > 16% had less than 0.04 g/dl CSF glucose. In the CSF of the eosinophil ≥ 10/mm^3^ group, out of 18 patients who underwent CSF culture or CSF bacterial PCR, 13 patients (72.2%) showed positive results: *Streptococcus suis* (n = 9), and *S*. *pneumoniae* (n = 4). In the CSF eosinophils ≥ 10% group, only 1 out of 7 patients (14.3%) showed a positive result of CSF bacterial PCR, which was *Neisseria meningitidis*. There was a significantly higher prevalence of CSF culture or bacterial-positive PCR results in the CSF eosinophil ≥ 10/mm^3^ group (OR 12.0 [95% CI 4.1; 34.8], p<0.001) compared with the non-EM/PM group.

Concerning the peripheral blood findings, both the median blood eosinophil percentages and eosinophil counts of the CSF eosinophil ≥ 10% group were lower than those of the blood eosinophil > 16% group. Patients with CSF eosinophils ≥ 10/mm^3^ had few eosinophils and increased numbers of neutrophils in their peripheral blood.

Regarding the serological test for the 4 parasites using blood samples, the samples were positive for only *A*. *cantonensis*. Of the 54 patients whose samples were tested by serology, 7 patients had positive results; 2 patients were from the CSF eosinophil ≥ 10% group, and 5 patients were from the blood eosinophil > 16% group. None of the 20 patients with CSF eosinophil counts ≥ 10/mm^3^ or the 20 control patients had any antiparasitic antibodies.

From the 34 (7, 20, and 7) patients fulfilling any of the three EM/PM definition criteria (criteria 1, 2 and 3, respectively), only 25 (5, 17, and 3) stored CSF samples were available. Of these samples, 3 were positive in the TaqMan Real-time PCR for *A*. *cantonensis*: 2 of the 5 CSF samples from the CSF eosinophil ≥ 10% group and 1 of 3 CSF samples from the blood eosinophil > 16% group. None of the 17 CSF samples from the CSF eosinophil ≥ 10/mm^3^ group was positive. None of the 25 CSF sample fulfilling any of the three EM/PM definition criteria and the 20 CSF sample from same control patients for the ELISA was positive in the SYBR Green Real-time PCR for *G*. *spinigerum*.

Table 2 shows a summary of each patient’s detail clinical information and the results of the serology and real-time PCR tests for *A*. *cantonensis* among the CSF eosinophil ≥ 10% and blood eosinophil > 16% groups. Only 1 patient with eosinophils ≥ 10% of the CSF WBCs showed both positive serology and real-time PCR results for *A*. *cantonensis*. The other 2 real-time PCR-positive patients had negative serological tests.

**Table 2:** Summary of the detailed information for each patient and the results of the serological tests and real-time PCR for *A*. *cantonensis* among the high CSF and blood eosinophilia groups.

	Age/ Sex	Chief complaint#	Duration of illness (days)	CSF cell absolute count /mm^3^ (Eosinophil% / Neutrophil% / Lymphocyte%)	Blood WBC absolute count /mm^3^ (Eosinophil%)	*A*. *cantonensis* serological test	*A*. *cantonensis* Real-time PCR	Detail clinical information
CSF eosinophils ≥ 10% group	33/M	Fever Headache	60	1070 (45.0 / 10.0 / 45.0)	5,500 (16.1)	Positive	ND	After oral Albendazole was prescribed, the symptoms disappeared. He was discharged without any neurologic sequelae after 2 weeks of treatment. However, CSF eosinophilia remained in the 10 days.
	21/M	Headache Left hemiplegia (Fever)	7	1420 (20.0 / 0 / 80.0)	10,280 (10.3)	Negative	Positive	He was treated with oral Albendazole, Ceftriaxone and Methylprednisolone. The left hemiplegia disappeared after 2 days and the headache subsided after 16 days of treatment. He was discharged with any neurologic sequelae after 20 days of hospitalization. However, CSF eosinophilia remained in the 2 weeks. CSF bacterial PCR also showed positive later.
	30/M	High fever Severe headache	2	2320 (64.2 / 17.7 / 18.1)	10,890 (7.1)	Negative	Negative	He was treated with oral Albendazole, Ceftriaxone and Methylprednisolone. High fever and headache subsided 2 days and 7 days after the treatments, respectively. Eosinophils in CSF disappeared after 5 days of treatment. He was discharged with any neurologic sequelae after 8 days of hospitalization.
	29/F	Joint pain Fever Headache	16	1380 (30.0 / 40.0 / 30.0)	9,230 (20.7)	Negative	Negative	She was treated with Albendazole, Ceftriaxone, and Methylprednisolone. After starting these medicines, her 6-week pregnancy was revealed and she was aborted. The symptoms generally improved. After 18 days of treatment, she was discharged without any sequelae.
	41/M	Fever Headache	20	1080 (40.0 / 20.0 / 40.0)	8,820 (30.3)	Negative	Negative	He underwent treatment with Albendazole and Methylprednisolone. After one week, CSF eosinophils disappeared. After ten days of treatment, the symptoms subsided and he was discharged with any sequelae.
	23/F	Fever Headache	7	2310 (58.4 / 3.5 / 32.9)	10,840 (27.1)	Negative	ND	She was treated with Albendazole and Methylprednisolone, and then the symptoms disappeared. She was discharged without any sequelae.
	18/M	Fever (Headache)	15	520 (50.0 / 40.0 / 10.0)	10,910 (18.4)	Positive	Positive	He initially underwent treatment with Doxycycline and Ceftriaxone. However, pain of lower legs, ascites, and skin congestion developed on day 2. On days 6 of hospitalization, the first CSF examination revealed eosinophilia, and EM was diagnosed. All antibiotics were stopped and replaced with Albendazole and steroids. The symptoms gradually improved.
Blood eosinophils > 16% group	59/M	(Fever, Headache)	7	460 (0.0 / 40.0 / 60.0)	11,940 (39.9)	Positive	ND	He was treated with antibiotics without Albendazole. No further clinical information was available.
	17 M	Headache Mild fever	5	350 (0.0 / 10.0 / 90.0)	16,500 (30.1)	Negative	Positive	He was initially treated with Ceftriaxone for one day. However, because eosinophil count of the peripheral blood increased despite did not have any allergy history. He was diagnosed as EM and prescribed Albendazole and steroid. Serology test for *Strongyloides* was negative. He did not have any allergy history and recovered fully after 8 days treatment. During his hospitalization, CSF data was followed up three times, and CSF eosinophilia was never confirmed.
	32/M	Headache Mild fever	10	540 (0.0 / 30.0 / 70.0)	18,000 (22.8)	Positive	ND	He was diagnosed with EM because of subacute meningitis symptom, rash, itch and peripheral blood eosinophilia. Albendazole and steroids were prescribed for 5 days. His symptoms disappeared gradually. During his hospitalization, CSF data was followed up three times, and CSF eosinophilia was never confirmed.
	28/M	(Fever, Headache)	5	420 (0.0 / 30.0 /70.0)	11,800 (29.2)	Positive	ND	He was treated with antibiotics without Albendazole. No further clinical information was available.
	32/M	(Fever, Headache)	10	310 (0.0 / 10.0 / 90.0)	6,880 (18.3)	Negative	ND	He was treated with antibiotics without Albendazole. No further clinical information was available.
	53/F	Headache (Fever)	25	860 (0.0 / 70.0 / 30.0)	9,670 (22.4)	Positive	Negative	She was hospitalized 5 days in Neurology department of Bach Mai Hospital. Peripheral blood eosinophilia was observed, and she did not have any allergy history. She was treated with ceftriaxone as bacterial meningitis for 10 days and recovered. During her hospitalization, CSF data was followed up two times, and CSF eosinophilia was never confirmed.
	39/M	(Fever, Headache)	25	140 (0.0 / 70.0 / 30.0)	9,120 (16.9)	Positive	Negative	Serological test for *Toxocara* was positive after hospitalization and Albendazole was stared. No further clinical information was available.

CSF: cerebrospinal fluid, EM/PM: eosinophilic meningitis or suspected parasitic meningitis, WBC: white blood cell, PCR: polymerase chain reaction, ND: Not Done.

# Bracket indicates symptom checked only in the study data base but not in the clinical record or if the clinical record was not available.

## Discussion

This prospective descriptive study included all patients with a suspected CNS infection at the largest referral medical center in Hanoi, northern Vietnam. This is the first study focusing on the implication of various EM definitions in the context of diagnosing PM. Our results indicate that the characteristics of patients with CSF eosinophil counts ≥ 10/mm^3^ but CSF eosinophil percentages < 10% were consistent with those of bacterial meningitis patients. Further serological and real-time PCR results indicated that there might be a non-negligible number of patients with PM without eosinophils in the CSF or fulfilling any of the previously defined EM criteria.

We defined these EM/PM criteria because first, the criteria of CSF eosinophils ≥ 10% was most commonly used, followed by the absolute number of eosinophils ≥ 10/mm3, and by peripheral blood eosinophils >16% in previous publications [9,8,15]. Second, we followed clinical diagnosis applied by the local clinicians at Bach Mai Hospital.

In our study, the prevalence of EM patients with CSF eosinophils accounting for ≥ 10% of the WBC count was 1.03% among total patients with a suspected CNS infection. This prevalence is higher than that previously reported in the southern and middle regions of Vietnam, which was 0.6% among 1241 CNS-infection patients [22] and 0.69% among 1000 CNS-infection patients aged ≥ 15 years without HIV [23], respectively. The prevalence of EM patients with CSF eosinophils ≥ 10% of the WBCs in our study was lower than that of the first report from northern Vietnam, which was 1.42% among 352 CNS-infection patients [24]. However, in this report, the definition of EM included the presence of ≥ 10 eosinophils/mm^3^ in addition to eosinophilia ≥ 10% of the WBCs in the CSF. If the same definition was applied herein, the prevalence would be 3.98%. These previous studies did not specifically discuss the differences in patient characteristics according to each EM definition.

In endemic areas, the majority of patients fulfilling the EM criteria are more likely to have a parasitic infection [1,4,5]. However, our results show that patients with eosinophils ≥ 10 cells/mm^3^ but not ≥ 10% of the WBCs in the CSF were more likely than those in the other groups to have bacterial meningitis because their clinical characteristics tended to present as acute, associated with a reduced level of consciousness, an increased number of neutrophils, an increased level of protein and a reduced level of glucose in the CSF. Therefore, the eosinophils ≥10 cells/mm^3^ criterion should be carefully interpreted. In fact, this EM criterion was not included in the diagnosis of EM caused by parasites in recent papers from Thailand, Vietnam and Laos [11–13,16,22].

Intriguingly, the clinical characteristics and laboratory results of patients with blood eosinophils accounting for > 16% of the WBCs were similar to those of EM patients with CSF eosinophils accounting for ≥ 10% of the WBCs, with the exception of CSF eosinophilia. None of the patients in this group had any eosinophils in their CSF, though they had an abnormally high number of cells in the CSF with a median of 420 (IQR 310–540) cells/mm^3^, which were predominantly neutrophils (n = 2) or lymphocytes (n = 5).

Our serological results identified 7 patients with antibodies against *A*. *cantonensis*: 2 (28.6%) patients from the CSF eosinophils ≥ 10% group and 5 (71.4%) patients from the blood eosinophils > 16% without CSF eosinophils group. In contrast, none of the 20 patients with CSF eosinophil counts ≥ 10/mm^3^ nor the 20 control patients had any anti-parasite antibodies. Subsequent real-time PCR analyses identified 3 patients positive for *A*. *cantonensis* and no patient positive for *G*. *spinigerum*, indicating that EM in these patients was due to *A*. *cantonensis*. Interestingly, one of the PCR-positive patients had blood eosinophils > 16% of the WBCs without CSF eosinophils. This finding, together with the highest seroprevalence among patients with blood eosinophils > 16% and their clinical characteristics being compatible with a parasitic infection, raises the hypothesis that patients in this group may have genuine PM. To date, many papers have reported that patients with *A*. *cantonensis*-induced EM have blood eosinophilia [2,10,20], and in a study setting where lumbar puncture was difficult to perform, blood eosinophilia alone was used to clinically diagnose patients with *A*. *cantonensis* infection-induced PM [14]. However, peripheral eosinophilia and serological tests should be cautiously used as a definitive evidence of PM. There have been no reports attempting to confirm *A*. *cantonensis* infection in clinically suspected patients with blood eosinophilia using real-time PCR. According to the previously published study [10,14], exposure history to *A*. *cantonensis* is an important clue to diagnose angiostrongyliasis. However, none of our patients positive for serology or real-time PCR mentioned exposure history.

According to a recent study of the pharmacodynamic effects of helminth-derived molecules using mouse models and soluble antigens of *A*. *cantonensis* larvae, an increase in blood eosinophil percentage was found to precede the CSF eosinophil percentage increase in mice, which are nonpermissive hosts, with a 14-day lag [25]. Although there has been no report describing exactly when CSF and blood eosinophil numbers begin to increase after infection with *A*. *cantonensis* in humans, it is plausible that there might be a lag between CSF and peripheral blood eosinophil responses among PM patients. In fact, the presence of low percentage or no eosinophils in the CSF during early stage of angiostrongyliasis has been previously reported [10,26]. Trevor J. Slom et al. reported that among 9 hospitalized patients with suspected EM caused by *A*. *cantonensis*, only 5 had CSF eosinophilia in the initial lumbar puncture [26], and 8 of the 9 patients finally exhibited CSF eosinophilia after hospitalization, although detailed changes in the patient blood eosinophil counts were not clearly reported. Furthermore, there has been a case report about a pediatric patient with *A*. *cantonensis*-induced EM within the USA [27]. His first CSF sample showed a WBC count of 763/mm^3^ with 5% eosinophils, but later his CSF showed high eosinophilia, reaching 21% of the CSF WBCs. However, to our knowledge, no study has performed both PCR and serology tests for diagnosing *A*. *cantonensis* on multiple meningitis patients without CSF eosinophils. In our study patients, three, whose CSF data were followed up, did not show CSF eosinophilia despite multiple lumbar punctures. However, at least 3 patients were prescribed albendazole during admission and reported significant clinical improvements, such as a reduction in headache severity.

The sensitivity and specificity of the current *A*. *cantonensis* ELISA and real-time PCR was not yet established. Development of serological diagnosis of helminth infection remains difficult. Several studies attempted to establish serological tests to diagnose *A*. *cantonensis* infection [28]. However, it is challenging to standardize parasitic meningitis with *A*. *cantonesis* because the presence of parasite bodies cannot be demonstrated in the majority of cases thus in most studies, positive cases were indirectly diagnosed by clinical symptoms and clinical histories [29–31]. Similarly, none of previously published studies with PCR have shown reliable data of sensitivity and specificity of PCR. McBride A *et al*. reported that 37 CSF samples (67.8%) was positive among 57 CSF samples of patients with CSF eosinophils ≥ 10%, using the same real-time PCR assay [23]. But this study did not show the result of non-EM/PM patients.

The main limitation of this study is that our study population biased toward febrile patients since the inclusion criteria included a history of fever anytime from onset to admission. Nevertheless, this was necessary in the current study as it primarily aimed to provide useful information to clinicians working in infectious disease wards to improve clinical judgment and management of patients suspected with meningitis. The latest literature review reported that around 45% of adult patients with EM/PM are afebrile [32], therefore our study population should be missing afebrile PM who did not have fever at any time point from onset to admission, and our findings be interpreted with caution and cannot be generalized to all patients with EM/PM. A community-based study approaching mildly symptomatic but infected individuals in the highly endemic area, and/or a hospital-based study including patients in the neurology department are warranted to reveal the whole clinical picture of PM/EM.

Our study has several other limitations. First, this was a single referral hospital-based study with a limited number of EM/PM patients. We could not have sufficient statistical power to demonstrate the significance of clinical features and sensitivity and specificity of the real-time PCR. However, Bach Mai Hospital is the biggest tertiary hospital in northern Vietnam and our study patients were identified as a consequence of screening a substantial number of patients with suspected CNS infections and CSF results. Collaborative studies are necessary for further investigation of such a rare clinical syndrome. A community-based study could provide a broader perspective on the epidemiology of EM/PM and a different prevalence of EM/PM. Since lumbar puncture was not required as a routine investigation for all patients with a headache, many mild cases of EM/PM may be treated as a nonspecific headache. Second, while general information was prospectively collected, the detailed clinical information of EM/PM patients was collected retrospectively from medical records. In particular, the information of three patients with high blood eosinophilia but CSF eosinophil percentage <10% was missing. Third, we used a serological test for *A*. *cantonensis* identification, which has not been validated in the context of northern Vietnamese population. We do not fully know the baseline sero-prevalence of people at a various degree of high risk of exposure to these parasites thus we need carefully interpret the positive results with this serology test. Forth, it is possible that patients with negative results in the real-time PCR or serological tests may have had CSF eosinophilia due to other infectious or noninfectious causes, which we did not aggressively investigate. However, regarding noninfectious causes, among the patients with CSF eosinophils ≥ 10% or blood eosinophils > 16% patients, any drug allergy was not confirmed, and neoplasms were unlikely because of their long-term clinical courses. Tuberculosis may also cause EM or even blood eosinophilia but none of EM/PM suspected patients was diagnosed as tuberculous meningitis as defined by PCR test with their CSF samples. One of patients with blood eosinophils > 16% group was positive *Toxocara spp*. serology in Bach Mai Hospital, whose serological test for *A*. *cantonensis* was also positive in this study. There is also a possibility of *G*. *spinigerum* infection because its infection in swamp eels and in human at Vietnam were reported [33,34] and this area is included in an endemic area of this parasite. However, none of patients with CSF eosinophil ≥ 10% or blood eosinophil > 16% accompanied its typical symptoms, such as swelling of the limbs, the trunk or the face with migration. None of their real-time PCR for *G*. *spinigerum* was positive. Furthermore, a possibility of false negative result of the real-time PCR test for *A*. *cantonensis* cannot be excluded due to insufficient sensitivity.

In conclusion, in northern Vietnam, the prevalence of EM was 1.03% among patients with a history of fever, suspected of having CNS infection if the definition of EM is eosinophils ≥ 10% of CSF WBC. Patients with CSF eosinophils ≥ 10/mm^3^ without high CSF eosinophilia more than 10% can be bacterial meningitis. Therefore, the percentage is more reliable than the absolute eosinophil count in the CSF for predicting PM. Despite the lack of exposure history to the parasite, clinical features, serology and real-time PCR suggest that the most likely etiology of EM seems to be *A*. *cantonensis* in this area. Our results re-confirmed previously reported findings that PM due to *A*. *cantonensis* infection may have CSF eosinophils less than 10% or even subsequent CSF eosinophils with no CSF eosinophils at the beginning. Diagnosing PM is challenging.

## Supporting information

S1 Strobe Checklist (DOC)

S1 Fig Relationships with each definitions of EM/PM case. (TIF)
