# Supplementary material for: Meningitis patients with Angiostrongylus cantonensis may present without eosinophilia in the cerebrospinal fluid in northern Vietnam
Source: PLoS Negl Trop Dis. 2020 Dec 22;14(12):e0008937. doi: 10.1371/journal.pntd.0008937 (PMC7810332; doi:10.1371/journal.pntd.0008937)
Supplement: S1 Strobe Checklist — (DOC) [file pntd.0008937.s001.doc]

STROBE Statement—checklist of items that should be included in reports of observational studies

|  | Item No | Recommendation | Section / paragraph | Relevant text from manuscript |
| --- | --- | --- | --- | --- |
| **Title and abstract** | 1 | (*a*) Indicate the study’s design with a commonly used term in the title or the abstract | Abstract | An observational study at a tertiary hospital. |
|  |  | (*b*) Provide in the abstract an informative and balanced summary of what was done and what was found | Abstract | Please read the abstract. |
| Introduction | | |  |  |
| Background/rationale | 2 | Explain the scientific background and rationale for the investigation being reported | 1^st^ – last paragraphs | The last sentence of the 1^st^ paragraph and the 1^st^ sentences of other three paragraphs. |
| Objectives | 3 | State specific objectives, including any prespecified hypotheses | Last paragraph | The 4^th^ and last sentences. |
| Methods | | |  |  |
| Study design | 4 | Present key elements of study design early in the paper | Study design and setting | The 1^st^ sentence of the section (a prospective observational study). |
| Setting | 5 | Describe the setting, locations, and relevant dates, including periods of recruitment, exposure, follow-up, and data collection | Study design and setting | Please read the paragraph. |
| Participants | 6 | (*a*) Give the eligibility criteria, and the sources and methods of selection of participants. | Inclusion and exclusion criteria and case definition | 1^st^-2^nd^ sentences of the 1^st^ paragraph of the section. |
| Variables | 7 | Clearly define all outcomes, exposures, predictors, potential confounders, and effect modifiers. Give diagnostic criteria, if applicable | Inclusion and exclusion criteria and case definition | 2^nd^ paragraph of the section. Please read the paragraph. |
| Data sources/ measurement | 8 | For each variable of interest, give sources of data and details of methods of assessment (measurement). Describe comparability of assessment methods if there is more than one group | Data and sample collection / Biological analysis / Statistical analysis | Please read the whole sections. All the clinical information was collected from medical charts, and the diagnostic test for *Angiostrongylus cantonensis* and other parasites were written in Biological analysis. |
| Bias | 9 | Describe any efforts to address potential sources of bias |  | Not applicable. |
| Study size | 10 | Explain how the study size was arrived at |  | Not estimated in advance. |
| Quantitative variables | 11 | Explain how quantitative variables were handled in the analyses. If applicable, describe which groupings were chosen and why | Statistical analysis | Whole the section describes how quantitative variables were handled. |
| Statistical methods | 12 | (*a*) Describe all statistical methods, including those used to control for confounding | Statistical analysis | Please read the section. |
|  |  | (*b*) Describe any methods used to examine subgroups and interactions |  | Not applicable. |
|  |  | (*c*) Explain how missing data were addressed |  | Not applicable. |
|  |  | (*d*) If applicable, describe analytical methods taking account of sampling strategy |  | Not applicable. |
|  |  | (*e*) Describe any sensitivity analyses |  | Not applicable. |

| Results | | |  | |  | |
| --- | --- | --- | --- | --- | --- | --- |
| Participants | 13 | (a) Report numbers of individuals at each stage of study—eg numbers potentially eligible, examined for eligibility, confirmed eligible, included in the study, completing follow-up, and analysed | | 1^st^-2^nd^ paragraphs, Fig 1 and S1 Fig | | Please read the two paragraphs of Results and see Fig 1 and S1 Fig. |
|  |  | (b) Give reasons for non-participation at each stage | |  | | Not applicable. |
|  |  | (c) Consider use of a flow diagram | | Fig 1 and S1 Fig. | | Please see Fig 1 and S1 Fig. |
| Descriptive data | 14 | (a) Give characteristics of study participants (eg demographic, clinical, social) and information on exposures and potential confounders | | 3^rd^-6^th^ paragraphs and Table 1 | | Please read whole the four paragraphs and see Table 1. |
|  |  | (b) Indicate number of participants with missing data for each variable of interest | | Table 1 | | Please see Table 1. |
| Outcome data | 15 | Report numbers of outcome events or summary measures | | 2^nd^ paragraph, Fig 1,Table 1 and Table 2 | | Please read the paragraph and see Fig 1, Table 1 and Table 2. |
| Main results | 16 | (*a*) Give unadjusted estimates and, if applicable, confounder-adjusted estimates and their precision (eg, 95% confidence interval). Make clear which confounders were adjusted for and why they were included | | 4^th^- 9^th^ paragraphs, Table 1 and Table 2. | | Please read the paragraphs and see Table 1 and Table 2. |
|  |  | (*b*) Report category boundaries when continuous variables were categorized | | Table 1 | | Please see Table 1. |
|  |  | (*c*) If relevant, consider translating estimates of relative risk into absolute risk for a meaningful time period | |  | | Not applicable. |
| Other analyses | 17 | Report other analyses done—eg analyses of subgroups and interactions, and sensitivity analyses | |  | | Not applicable. |
| Discussion | | |  | |  | |
| Key results | 18 | Summarise key results with reference to study objectives | | 1^st^ and last paragraphs | | Please read the 1^st^ and last paragraphs of Discussion. |
| Limitations | 19 | Discuss limitations of the study, taking into account sources of potential bias or imprecision. Discuss both direction and magnitude of any potential bias | | 9^th^ and 10^th^ paragraph | | Please read the 9^th^ and 10^th^ paragraph. |
| Interpretation | 20 | Give a cautious overall interpretation of results considering objectives, limitations, multiplicity of analyses, results from similar studies, and other relevant evidence | | 3^nd^-8^th^ paragraphs | | Please read whole the six paragraphs. |
| Generalisability | 21 | Discuss the generalisability (external validity) of the study results | | 4^th^ and 6^th^ paragraph | | Please read the 3^th^ to 4^th^ sentence of 4^th^ paragraph and the 3^rd^ to 7^th^ sentence of the 6^th^ paragraph. |
| Other information | | |  | |  | |
| Funding | 22 | Give the source of funding and the role of the funders for the present study and, if applicable, for the original study on which the present article is based | | Acknowledgement | | The 2^nd^ sentence of the section. |

*Give information separately for cases and controls in case-control studies and, if applicable, for exposed and unexposed groups in cohort and cross-sectional studies.

**Note:** An Explanation and Elaboration article discusses each checklist item and gives methodological background and published examples of transparent reporting. The STROBE checklist is best used in conjunction with this article (freely available on the Web sites of PLoS Medicine at http://www.plosmedicine.org/, Annals of Internal Medicine at http://www.annals.org/, and Epidemiology at http://www.epidem.com/). Information on the STROBE Initiative is available at www.strobe-statement.org.
