# Supplementary figures and images for: Meningitis patients with Angiostrongylus cantonensis may present without eosinophilia in the cerebrospinal fluid in northern Vietnam
Source: PLoS Negl Trop Dis. 2020 Dec 22;14(12):e0008937. doi: 10.1371/journal.pntd.0008937 (PMC7810332; doi:10.1371/journal.pntd.0008937)

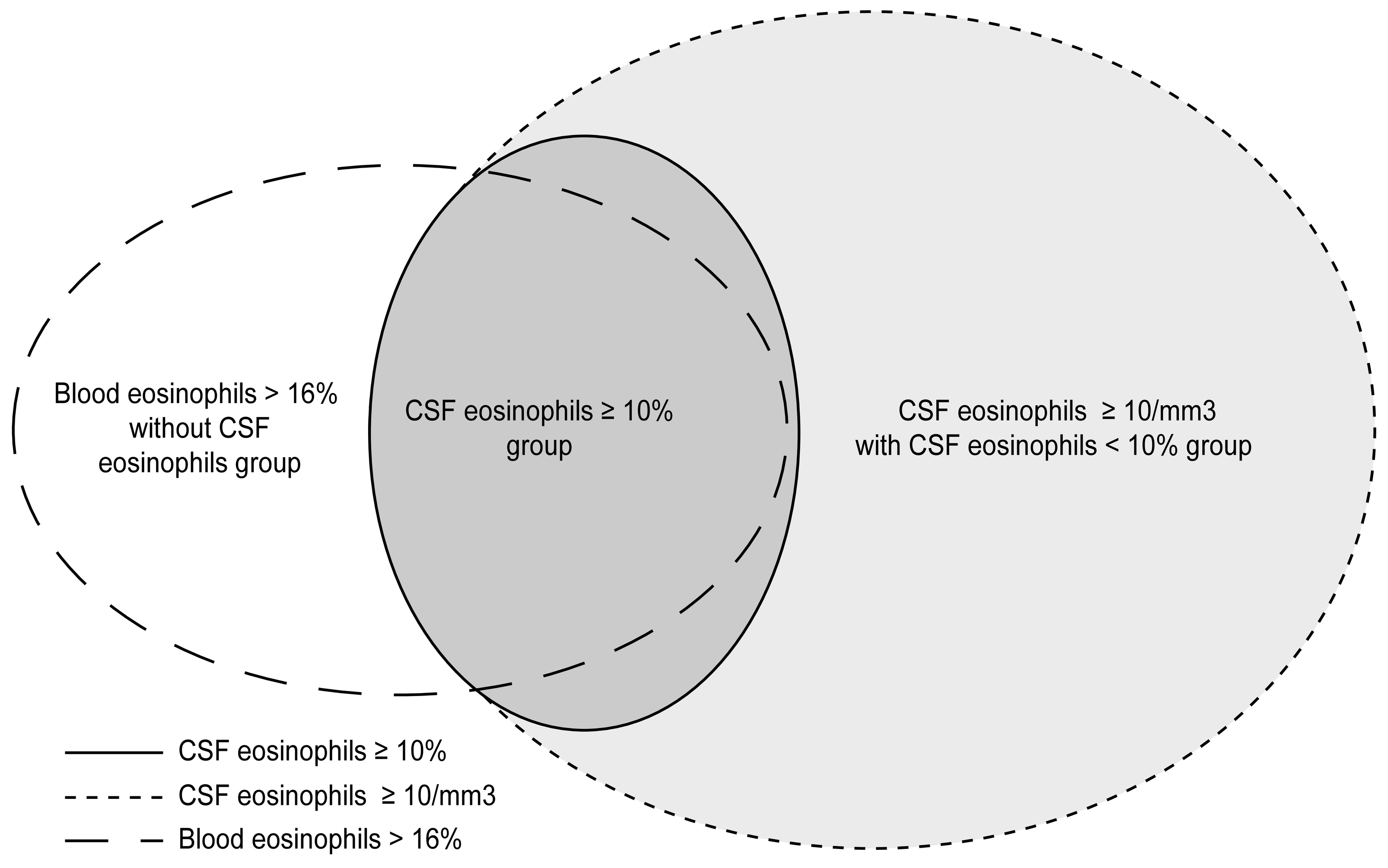

Supplement: S1 Fig — (TIF) [file pntd.0008937.s002.tif]
